# Nomogram Based on A‐To‐I RNA Editing for Predicting Overall Survival in Patients With Breast Cancer

**DOI:** 10.1111/jcmm.70781

**Published:** 2025-09-25

**Authors:** Yangyang Zhang, Jia Zhou, Hong Li, Dahai Chai, Bin Lian, Yaobang Liu, Li Guo, Jinping Li

**Affiliations:** ^1^ General Hospital of Ningxia Medical University Yinchuan Ningxia China; ^2^ Ningxia Hui Autonomous Region People's Hospital Yinchuan Ningxia China; ^3^ Department of Surgical Oncology General Hospital of Ningxia Medical University Yinchuan Ningxia China

**Keywords:** ATIRE, breast cancer, nomogram, overall survival, risk score

## Abstract

Adenosine‐to‐inosine RNA editing (ATIRE) is the most common type of RNA editing in higher eukaryotes. Many RNA editing events are associated with the occurrence and development of various tumours. Currently, several ATIRE sites have been used as predictors of cancer prognosis. However, whether some of them can be used as diagnostic and prognostic markers for patients with breast cancer (BRCA) remains unknown. BRCA‐related data and RNA editing data were downloaded from the TCGA database, and the patients were randomly divided into training (*n* = 503) and validation (*n* = 334) groups. Through univariate Cox regression, Lasso regression, and multivariate Cox regression analysis, nine ATIRE sites related to prognosis in all groups were identified to construct a prognostic model and generate an ATIRE risk score. The median survival time of patients with high‐risk scores was significantly shortened, and the nomogram performed well in predicting the overall survival time of patients with BRCA. Calibration and decision curves verified the high accuracy of the model. Among them, five ATIRE sites correlated with the expression of the corresponding genes, and the expression of four ATIRE sites in tumour tissues was significantly higher than that in normal tissues (*p* < 0.05). Reverse transcription quantitative polymerase chain reaction and immunohistochemical staining experiments were used for preliminary experimental validation of the results. The prognostic model based on ATIRE could serve as a new tool for predicting the survival and prognosis of patients with BRCA and help clinicians provide better individualised clinical decision‐making.

## 1 Introduction

According to global cancer data statistics for 2020, the number of new cases of breast cancer (BRCA) is 2.26 million, thus replacing lung cancer as the most common malignant tumour worldwide and demonstrating a tendency of rejuvenation. The number of cancer‐related deaths amongst women ranks first worldwide [1]. The systemic treatment of BRCA has initially formed a mature medical system, including surgery, chemotherapy, targeted therapy, radiotherapy and immunotherapy, and the treatment plan is more precise and personalised. However, owing to the highly heterogeneous nature of BRCA and its complex biological characteristics, treatment outcomes remain unsatisfactory. Therefore, the search for new prognostic markers and therapeutic targets is urgently required. With the development of medical technology, big data have been analysed for the diagnosis, treatment, and prognosis of various diseases. The establishment and application of prediction models provide important support for disease screening, diagnosis, treatment, and prognostic evaluation.

RNA editing refers to the biological phenomenon in which nucleotide insertion, deletion, or substitution occurs at the post‐transcriptional level, resulting in changes in the nucleotide sequence. Thus, the amino acid sequence, structure, function, or expression level of the translated protein is different from the genetic information carried by the original gene sequence. RNA editing is more tumour‐specific than gene expression [2], and is not affected by the number of isolated RNA or individual differences in reference gene selection, which makes it potentially useful for predicting prognosis and potential therapeutic sites. Adenosine‐to‐inosine RNA editing (A‐to‐I RNA editing, ATIRE) is the most common form of RNA editing that affects most human genes [3]. RNA editing plays an important regulatory role in tumour and immune processes [4]. Several tumour types show significant increases in RNA editing levels compared to normal tissues [5], some of which may be carcinogenic mutations, indicating that they serve as new candidates with therapeutic and diagnostic potential. In this study, we developed a predictive model for applying ATIRE to predict overall survival (OS) in patients with BRCA based on the Cancer Genome Atlas (TCGA) database and preliminarily explored the potential mechanism by which these ATIRE sites affect the prognosis of patients with BRCA.

## 2 Data and Methods

### 2.1 Downloading and Organising Data

**FIGURE 1:**
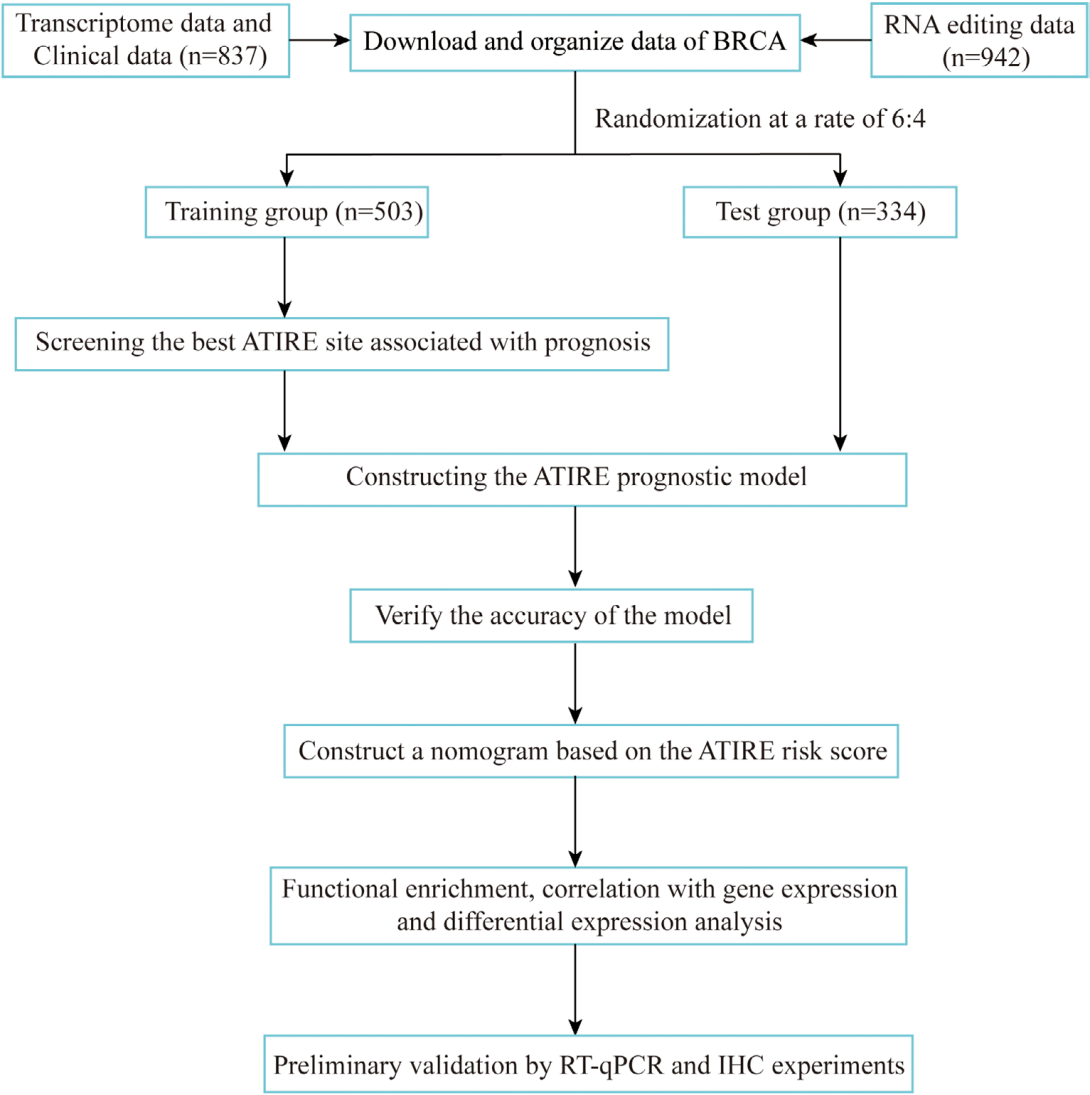
Technical route of screening key ATIRE and constructing a prognostic model.

Figure 1 shows the entire data processing procedure. Transcriptome data (*N* = 113, *T* = 1118) and clinically relevant data of BRCA normal and tumour tissues were downloaded from the TCGA database (https://portal.gdc.cancer.gov/). RNA editing data for the BRCA samples were downloaded from the Synapse website (https://www.synapse.org/). RNA editing data with missing values > 30% were deleted using PERL software, and a total of 942 samples with available ATIRE data were collected. Then the R package ‘impute’ was used to complete the missing values, and more than 90% of the rows with values < 0.05 were deleted. After removing the normal samples, 837 BRCA samples were included in the analysis. The obtained RNA editing data and extracted survival data were overlapped and merged.

### 2.2 Constructing a Prognostic Model

The createDataPartition function in the R software was used to randomly divide the samples into training (*n* = 503) and validation (*n* = 334) groups in a 6:4 ratio. First, univariate Cox regression analysis was performed on the training data to obtain prognosis‐related RNA editing in patients with BC. Then, the R package ‘glmnet’ was applied to perform the least absolute shrinkage and selection operator (LASSO) regression algorithm to determine the optimal prognostic ATIRE sites for the construction of the subsequent model, and the penalty parameter adjustment was performed through ten‐fold cross‐validation. Finally, a risk model was developed using multivariate Cox proportional risk regression analysis and the following model equation was obtained:

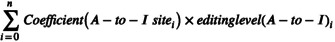




The risk score for each sample in the training group was calculated using a model formula. The samples were categorised into high‐ and low‐risk groups based on the median value (cut‐off = 50%) of the risk score, and Kaplan–Meier (KM) survival curves were plotted to compare the difference in survival time between the two groups.

### 2.3 Verifying the Accuracy of the Model

Samples were sorted according to the patients' risk scores, and a risk curve was drawn (including a risk score distribution curve graph, survival state scatter plot, and risk heat map). Independent prognostic and clinical correlation analyses were performed using univariate and multivariate Cox risk regression, incorporating the patients' clinical information (age, sex, clinical stage and tumour–node–metastasis [TNM] stage) with the risk scores obtained above. The R package ‘rms’ was used to establish a nomogram to predict the 1‐, 2‐, and 3‐year OS of patients with BRCA. Calibration, receiver operating characteristic (ROC), and decision curve analysis (DCA) were used to test the predictive efficacy of the combined model for consistency with reality.

### 2.4 Enrichment Analysis of Differential Genes and Functions

After removing the normal tissue samples from the expression data and taking the intersection with the risk file, samples from the low‐ and high‐risk groups were extracted for differential gene analysis. The filtering conditions were set to logFC = 0.585, *p*‐value = 0.05, FDR = 2 and *q*‐value = 0.05; significant differentially expressed genes (DEGs) were obtained. Enrichment analysis was performed using DEGs, including gene ontology (GO), Kyoto encyclopedia of genes and genomes (KEGG), gene set enrichment analysis (GSEA), and HALLMARK pathway.

### 2.5 Gene Expression Correlation and Differential Expression Analysis

The correlation between RNA editing and the corresponding gene expression, the correlation between risk score and ADAR expression, and the differential expression of selected ATIRE site editing levels in tumour and normal tissues were analysed.

### 2.6 Cell Culture and Acquisition of Clinical Samples

The normal mammary epithelial cell line MCF‐10A and BRCA cell lines MCF‐7, T47D, BT20 and MDA‐MB‐231 were purchased from the American Type Culture Collection. All cells were cultured in their respective media and incubated in a humidified incubator at 37°C with 5% CO_2_.

The medium components required for different cell lines are as follows:

**Table:** 

MCF‐10A cells: Basal medium +5% horse serum +20 ng/mL epidermal growth factor (EGF) + 1% penicillin/streptomycin (P/S).
T47D cells: RPMI‐1640 + 10% fetal bovine serum (FBS) + 10 μg/mL insulin +1% P/S.
MCF‐7, BT20 and MDA‐MB‐231 cells: DMEM complete medium +10% FBS + 1% P/S.

Forty pairs of BRCA and adjacent normal tissues were collected from patients who received surgery at the Department of Surgical Oncology, General Hospital of Ningxia Medical University between October 2024 and May 2025. None of the patients had received antitumor treatment preoperatively, including radiotherapy, chemotherapy, or immunotherapy. Fresh tissues were immediately stored at −80°C after resection. This study was approved by the Medical Research Ethics Committee of the General Hospital of Ningxia Medical University, and all patients provided signed informed consent prior to surgery.

### 2.7 Reverse Transcription Quantitative Polymerase Chain Reaction

Total RNA was extracted from BRCA cells using TRIzol reagent (Invitrogen). Subsequently, 1 μg of total RNA was reverse‐transcribed into cDNA using a HiScript III All‐in‐one RT SuperMix Perfect qPCR kit (Vazyme). Reverse Transcription Quantitative Polymerase Chain Reaction (RT‐qPCR) was performed using the SYBR Green PCR Master Mix with specifically designed primer sequences. Glyceraldehyde‐3‐phosphate dehydrogenase was set as the internal reference gene, and the relative expression level of the target gene was calculated using the 2^−∆∆Ct^ method. All experiments were repeated thrice. Primer sequences were designed and synthesised by Sangon Biotech (Shanghai, China). The following primer sequences were used:

**Table:** 

Primers	Sequences
ATXN3	Forward: 5′‐AGGGTCCAACAGATGCATCG‐3′ Reverse: 5′‐GACTTAGTGCCAGAGCCCTC‐3′
METTL2A	Forward: 5′‐CTTCTGAAACCTGGCGGGAT‐3′ Reverse: 5′‐AGCGTGTCCAGTTCCTCTTG‐3′
PEX26	Forward: 5′‐GTCCTGGAGCTGTGCATTCT‐3′ Reverse: 5′‐CTCCGATAAGCAGCCCAGAG‐3′
UGGT1	Forward: 5′‐AAGGATCATTGGGCCACTGG‐3′ Reverse: 5′‐TACCAAGTCGCTTGCCACAT‐3′
GAPDH	Forward: 5′‐GTCAACGGATTTGGTCTGTATT‐3′ Reverse: 5′‐AGTCTTCTGGGTGGCAGTGAT‐3′

### 2.8 Immunohistochemical Staining

The paraffin‐embedded tissues were cut into 4–5‐μm thick sections. After dewaxing, hydration, and antigen repair, the endogenous peroxidase was inactivated by 3% H_2_O_2_ treatment for 8 min and then incubated with 3% BSA blocking solution at room temperature for 30 min. After the sections were incubated with the corresponding primary antibody at 4°C overnight, the sections were incubated with the secondary antibody at room temperature for 1 h. The sections were stained with freshly prepared DAB solution, re‐stained with haematoxylin, and observed under a microscope. The following primary antibodies were used: ATXN3 (Cat No. 13505–1‐AP, 1:200), METTL2A (Cat No. 16983–1‐AP, 1:500), PEX26 (Cat No. 27472–1‐AP, 1:500) and UGGT1 (Cat No. 14170–1‐AP, 1:500). All the antibodies were purchased from Proteintech (Wuhan, China).

### 2.9 Statistical Analysis

The R language statistical software (version 4.4.1) was used. The Wilcoxon rank‐sum test was used for difference analysis, and Pearson correlation was used for correlation analysis. Statistical significance was set at *p* < 0.05. The RT‐qPCR results are presented as mean ± standard deviation. Differences between groups were analysed using a one‐way analysis of variance, with *p* < 0.05 considered statistically significant. GraphPad Prism 8 was used as the mapping software.

## 3 Results

### 3.1 Baseline Data of BRCA in TCGA Database

No significant differences in age, sex, grade, or TM stage were observed between the training and validation sets, except for N stage (*p* > 0.05); the baseline characteristics of the two groups were comparable (Table 1).

**TABLE 1:** Baseline data of BRCA in TCGA database.

Covariates	Type	Total	Test	Train	*p*
Age					0.0828
	<= 65	600 (71.68%)	251 (75.15%)	349 (69.38%)	
	> 65	237 (28.32%)	83 (24.85%)	154 (30.62%)	
Sex					0.4551
	Female	828 (98.92%)	332 (99.4%)	496 (98.61%)	
	Male	9 (1.08%)	2 (0.6%)	7 (1.39%)	
Stage					0
	Stage I–II	814 (97.25%)	323 (96.71%)	491 (97.61%)	
	Unknown	23 (2.75%)	11 (3.29%)	12 (2.39%)	
*T*					0.4629
	T1–2	715 (85.42%)	281 (84.13%)	434 (86.28%)	
	T3–4	120 (14.34%)	52 (15.57%)	68 (13.52%)	
	Unknown	2 (0.24%)	1 (0.3%)	1 (0.2%)	
*M*					0.9554
	M0	750 (89.61%)	299 (89.52%)	451 (89.66%)	
	M1	16 (1.91%)	7 (2.1%)	9 (1.79%)	
	Unknown	71 (8.48%)	28 (8.38%)	43 (8.55%)	
*N*					0.0088
	N0	393 (46.95%)	137 (41.02%)	256 (50.89%)	
	N1–3	429 (51.25%)	189 (56.59%)	240 (47.71%)	
	Unknown	15 (1.79%)	8 (2.4%)	7 (1.39%)	

### 3.2 Preliminary Screening for Prognostic Related ATIRE Sites

**FIGURE 2:**
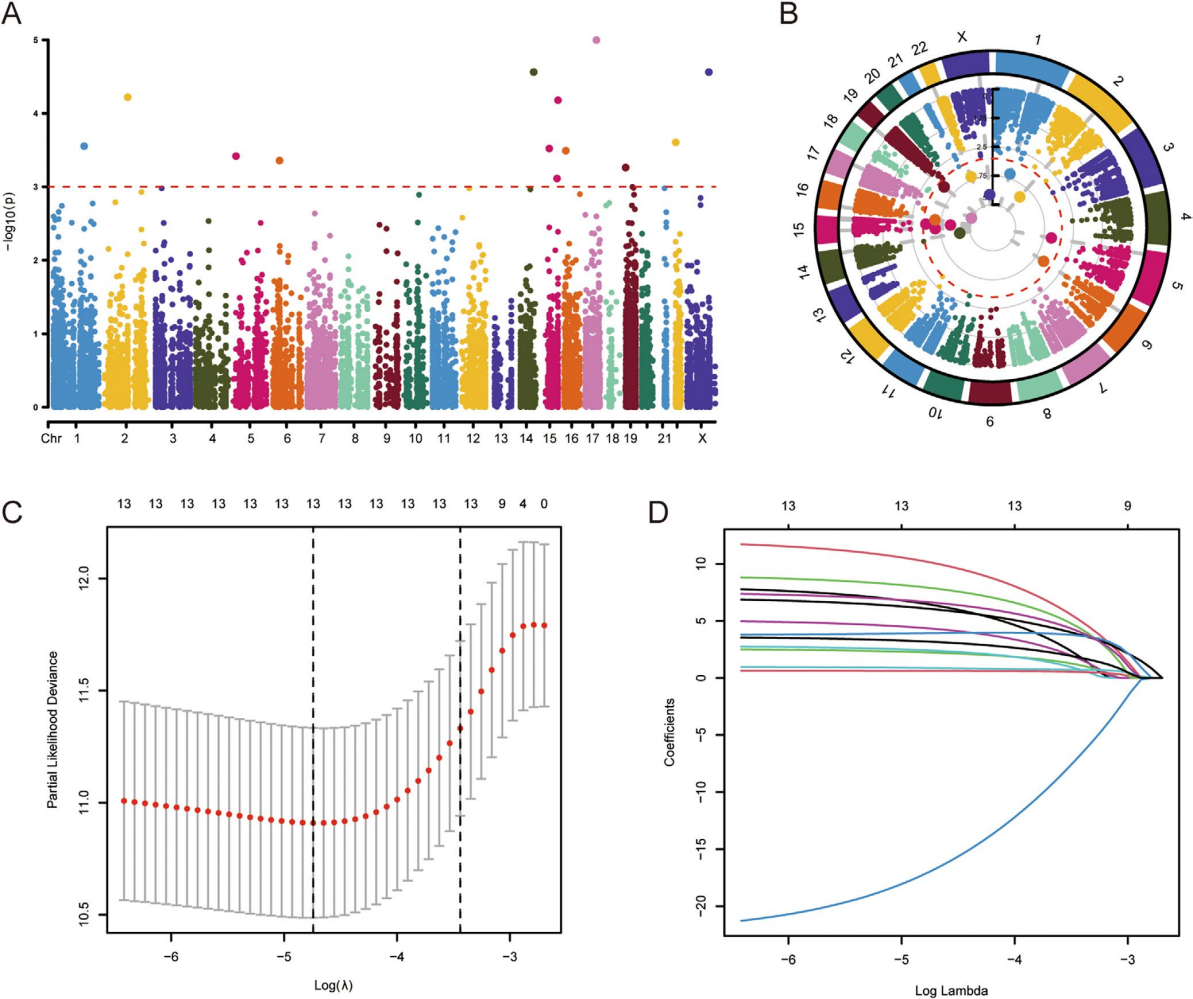
Identification of survival‐related ATIRE sites in patients with BRCA. (A) Manhattan plot showing ATIRE sites significantly associated with prognosis using *p* < 0.001 as a screening criterion. Where the x‐axis is the *p*‐value of prognostically associated ATIRE and the y‐axis is the chromosomal location of the ATIRE. (B) The circle diagram also shows the ATIRE sites related to prognosis. The outermost circle is the chromosome position of the ATIRE sites, and the inner point is the result of the ATIRE prognosis. (C) Selection of optimal parameters in the Lasso model by 10‐fold cross‐validation. (D) LASSO regression to determine the best prognostic ATIRE sites.

Through univariate Cox regression analysis of the training group, 13 ATIRE sites were identified to be significantly associated with the OS of patients with BRCA (*p* < 0.001), and the prognostic results were visualised to obtain Manhattan and circle diagrams (Figure 2A,B). The LASSO regression algorithm was used to avoid data overfitting, and the ATIRE corresponding to the point with the smallest error in the cross‐verification results was the final characteristic ATIRE site (Figure 2C,D). Multivariate Cox analysis identified nine sites as the best prognostic sites for subsequent model construction, and the following model formula was obtained to calculate the risk scores: (7.96 × ZNF554|chr19:2835122) + (13.72 × UGGT1|chr2:128949284) + (−23.44 × SCAND2P|chr15:85185369) + (4.26 × LRRC57|chr15:42835509) + (7.57 × METTL2A|chr17:60528133) + (10.54 × PEX26|chr22:18572607) + (6.83 × CXorf56|chrX:118672581) + (8.79 × ATXN3|chr14:92526779) + (3.91 × LOC100130744|chr5:14716392).

### 3.3 Model Construction and Preliminary Verification of Accuracy

**FIGURE 3:**
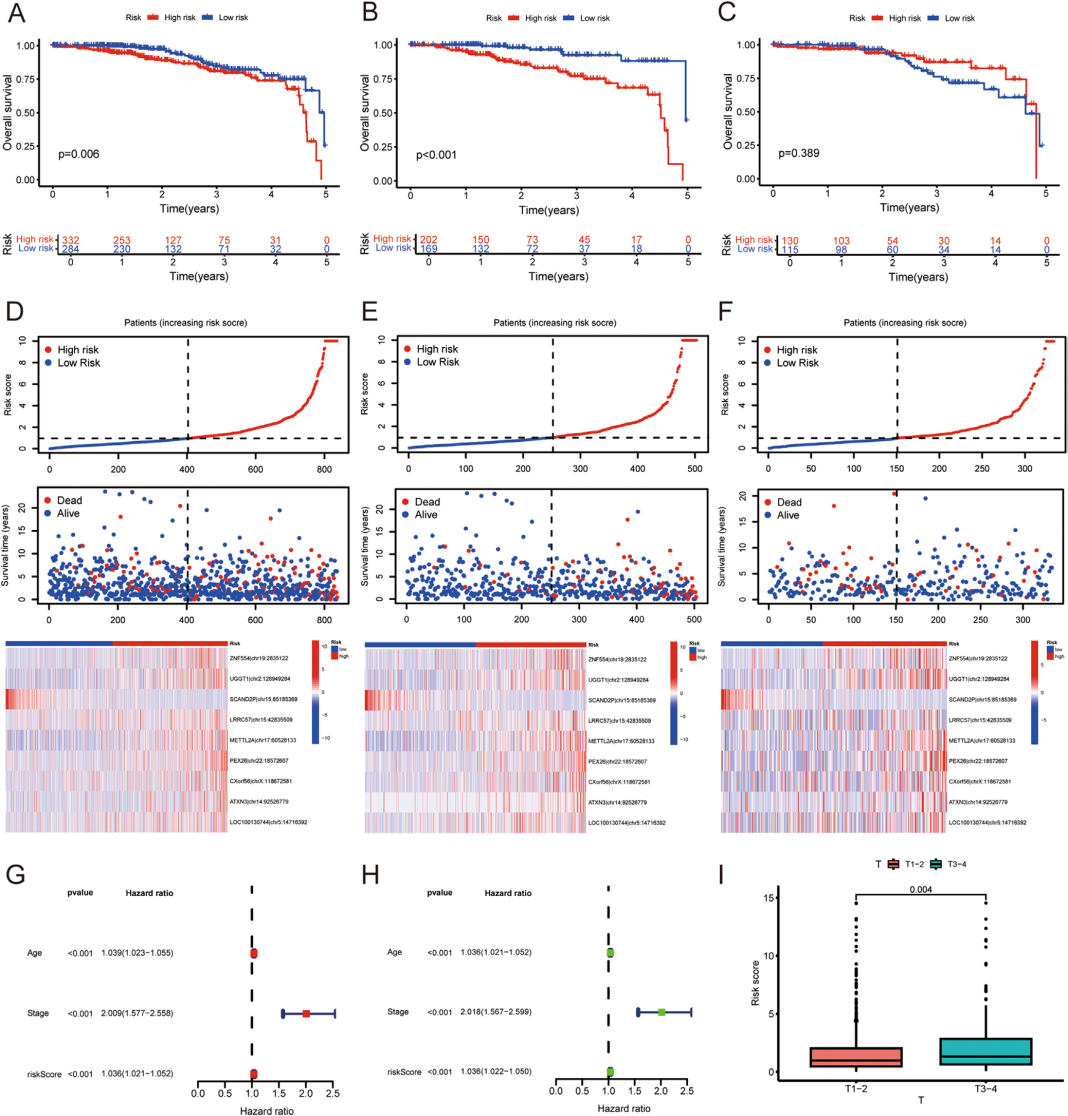
The relationship between ATIRE risk score and prognosis of patients with BRCA. (A–C) Visualised Kaplan–Meier plots of OS grouped by risk score in all patients (A), training group (B), and validation group (C). (D–F) The distribution of ATIRE risk score, survival status, and editing level of 9 ATIRE sites in all patients (D), training group (E), and validation group (F). G, H Multivariate (G) and univariate (H) Cox regression were used for independent prognostic analysis. (I) Clinical correlation analysis.

The samples were categorised into high‐ and low‐risk groups based on the median risk score. After analysing the survival differences between the samples, the OS of the high‐risk group in all samples and the training group was significantly lower than that of the low‐risk group (*p* < 0.05) (Figure 3A–C). Survival analysis was performed using the constructed RNA editing model, including a risk curve, survival status, and risk heat map. The mortality rate of BRCA in the high‐risk group was higher than in the low‐risk group. Except for SCAND2P | chr15:85185369, which was highly expressed in the low‐risk group, the remaining eight RNA‐editing sites were highly expressed in the high‐risk group (Figure 3D–F). The independent prognostic analysis revealed that the model based on ATIRE could be used as a prognostic factor independent of other clinical traits (Figure 3G,H). Further clinical correlation analysis revealed that the risk score was statistically significant only between stages T1–2 and T3–4 (Figure 3I).

### 3.4 Construction and Performance Evaluation of Nomogram

**FIGURE 4:**
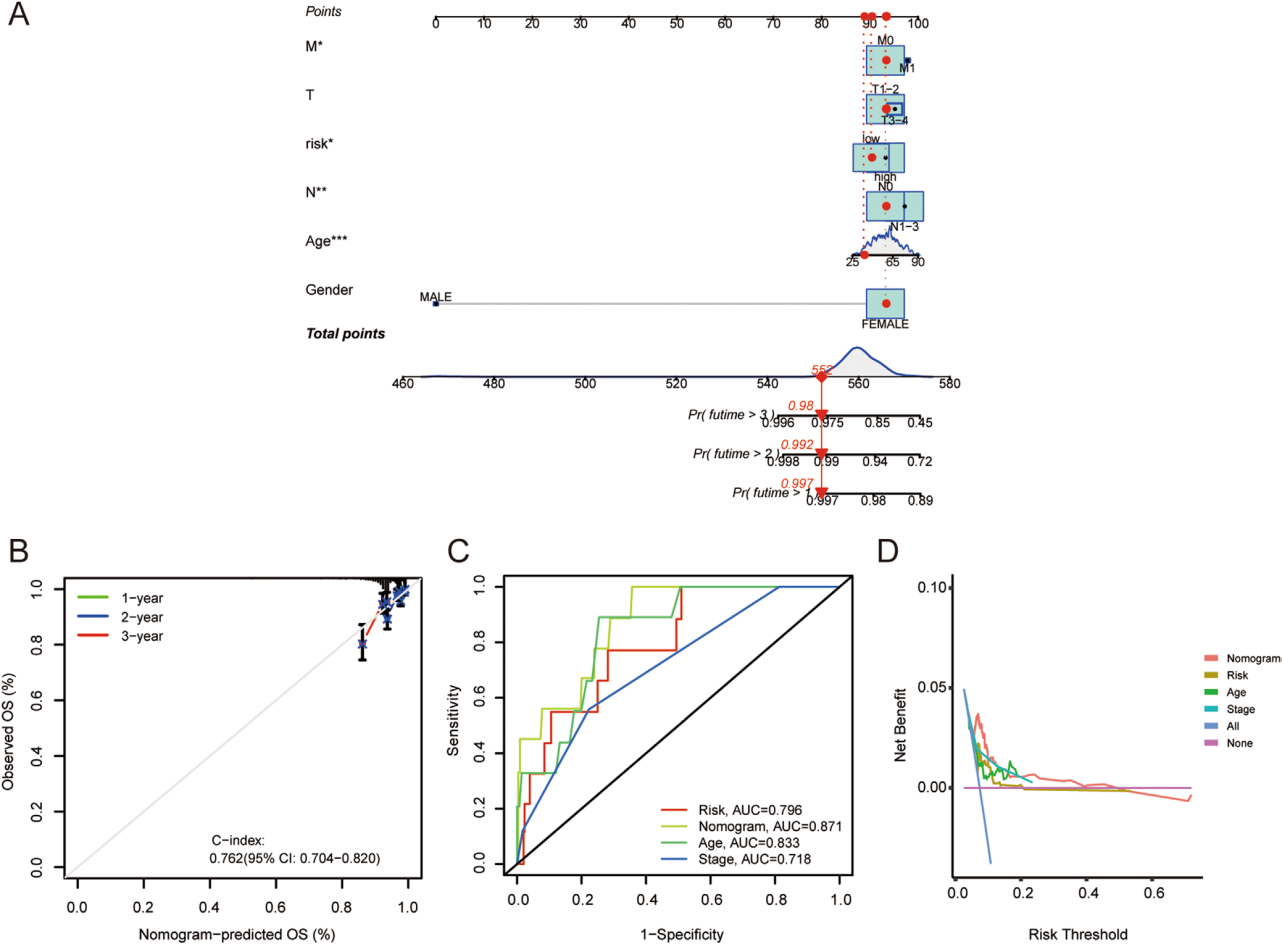
Construction and evaluation of nomogram. (A) Incorporation of ATIRE risk score and clinical parameters to construct a nomogram to predict the 1‐, 2‐ and 3‐year OS of patients with BRCA. (B) The OS observed by the calibration curve at 1, 2, and 3 years was basically consistent with the prediction of the nomogram. (C, D) ROC curve (C) and DCA curve (D) were used to test the clinical benefits of risk model and nomogram.

The ATIRE risk score and clinicopathological features including age, sex, clinical stage, and TNM stage were used to construct a nomogram (Figure 4A). The calibration curve (95% confidence interval [CI]: 0.704 to 0.820) showed a high degree of concordance (C‐index = 0.762) between the OS rates observed at 1‐year, 2‐year, and 3‐year and that predicted by the nomogram (Figure 4B). The ROC plots showed area under the curve (AUC) values of 0.796 and 0.871 for the constructed model and nomogram, respectively, which were greater than the AUC values of the two single clinical traits of age and stage (Figure 4C). In addition, DCA showed that the constructed model and nomogram had a higher net benefit than individual clinical‐pathology characteristics (Figure 4D).

### 3.5 Risk Difference and Functional Enrichment Analyses

**FIGURE 5:**
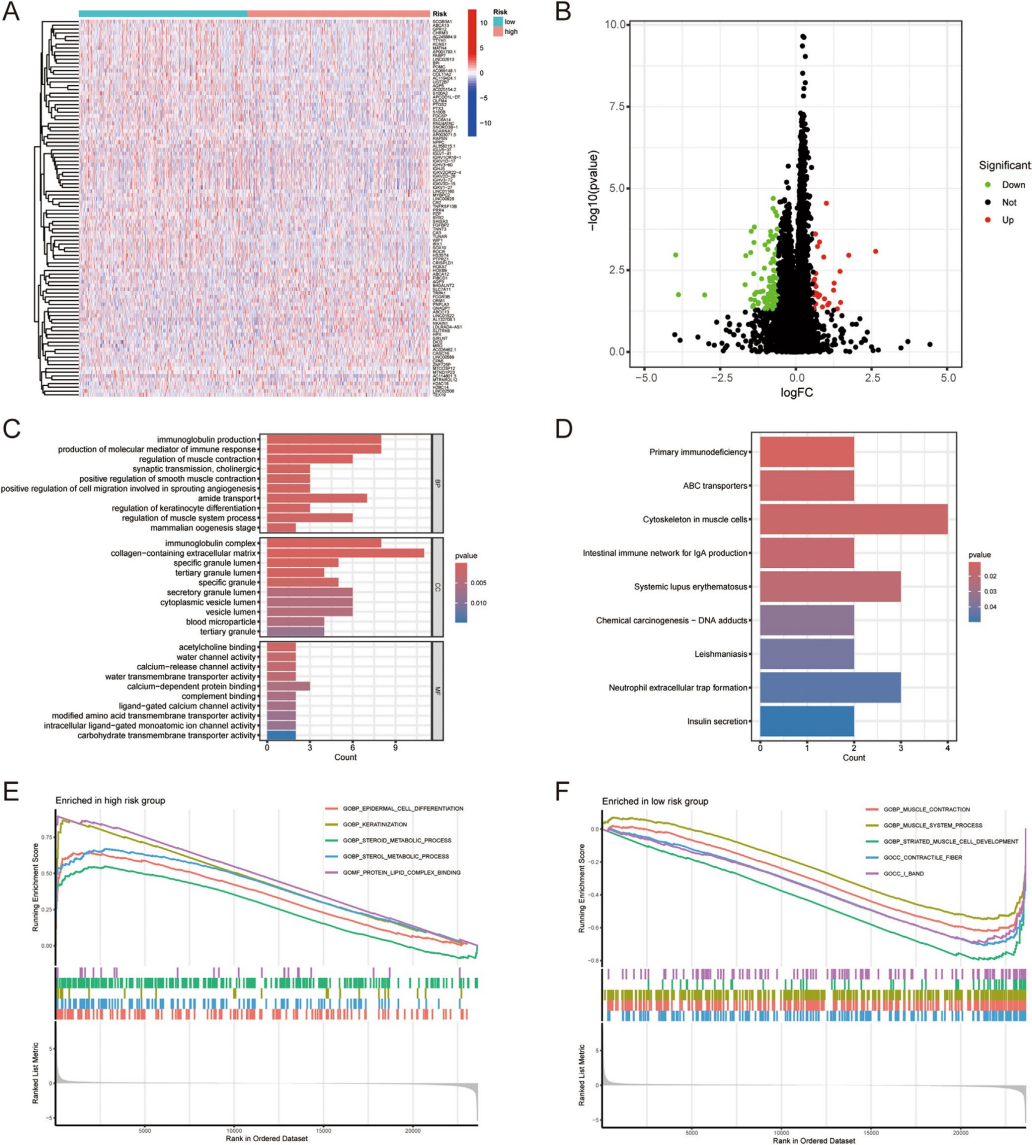
Screening DEGs in high and low risk groups and their functional enrichment analysis. (A, B) Heat map and volcano map of DEGs in high and low risk groups. (C) GO functional enrichment analysis. (D) KEGG functional enrichment analysis. (E, F) GSEA functional enrichment analysis.

Screening for DEGs in the high‐ and low‐risk groups showed that 30 genes were upregulated in the high‐risk group and 106 genes were upregulated in the low‐risk group (Figure 5A,B). GO functional enrichment analysis showed that the DEGs were mainly enriched in pathways such as immunoglobulin production, production of molecular mediators of the immune response, immunoglobulin complexes, collagen‐containing extracellular matrix, and acetylcholine binding (Figure 5C). KEGG functional enrichment analysis indicated that the DEGs mainly affected pathways such as primary immunodeficiency, ABC transporters, and the cytoskeleton in muscle cells (Figure 5D). GSEA functional enrichment analysis showed that functions and pathways such as epidermal cell differentiation, keratinization, steroid metabolism, sterol metabolism, and protein–lipid complex binding were concentrated in the high‐risk group, whereas muscle contraction, muscle system processes, striatum muscle cell development, contractile fibre, and I band were concentrated in the low‐risk group (Figure 5E,F). HALLMARK pathway analysis showed that the genes HALLMARK_INTERFERON_ALPHA_RESPONS and HALLMARK_INTERFERON_GAMMA_RESPON pathway (Figure S1).

### 3.6 Correlation Analysis of ATIRE and Corresponding Gene Expression

**FIGURE 6:**
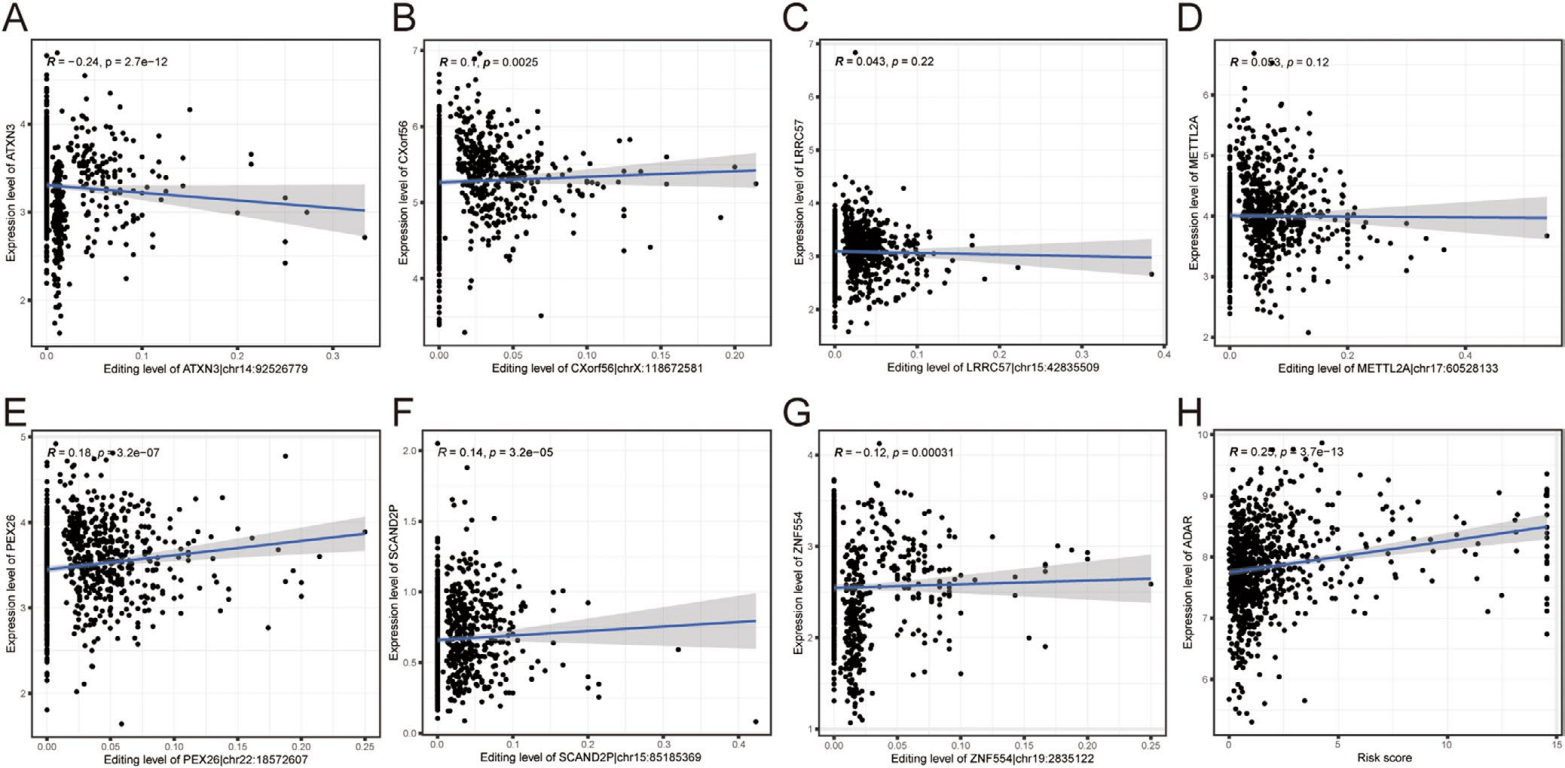
RNA editing correlation analysis. (A–G) Correlation analysis between RNA editing degree and corresponding gene expression level. (H) Correlation analysis between ADAR expression and risk score.

The degree of RNA editing of ATXN3 and ZNF554 was negatively correlated with the expression of the corresponding genes; a positive correlation was identified between the degree of RNA editing and the expression of the corresponding genes in CXorf56, PEX26 and SCAND2P, and the difference was statistically significant (*p* < 0.05) (Figure 6A–G). A significant positive correlation was identified between ADAR expression in BRCA and the risk score (*p* < 0.05) (Figure 6H).

### 3.7 Analysis of Differences in ATIRE Expression

**FIGURE 7:**
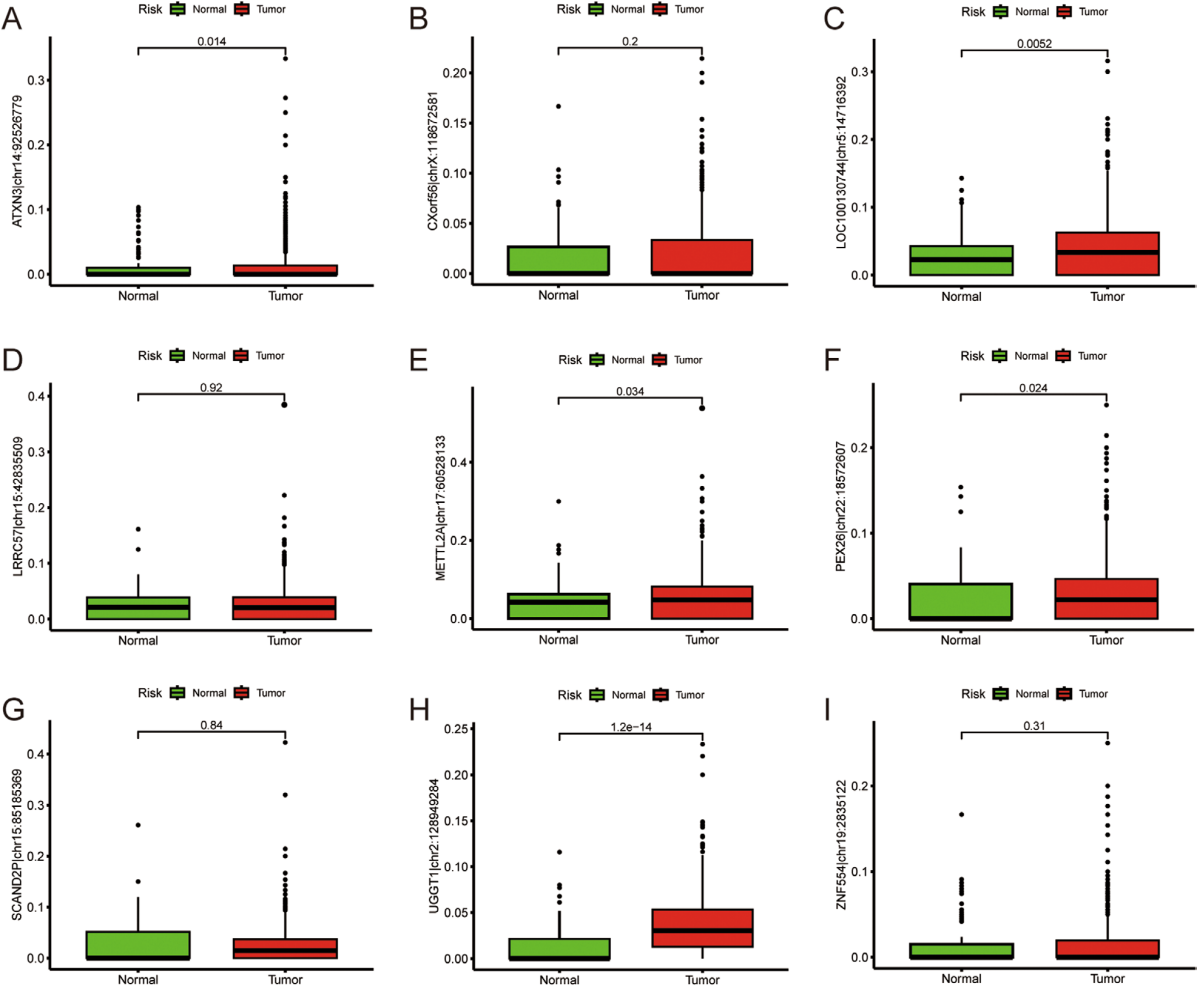
Differential expression analysis. (A–I) The differential expression of selected ATIRE sites in tumour tissues and normal tissues.

The expression levels of ATXN3 | chr14:92526779, LOC100130744 | chr5:14716392, METTL2A | chr17:60528133, PEX26 | chr22:18572607 and UGGT1 | chr2:128949284 in tumour tissues were significantly higher than those in normal tissues (*p* < 0.05) (Figure 7A–I).

### 3.8 Reverse Transcription Quantitative Polymerase Chain Reaction

**FIGURE 8:**
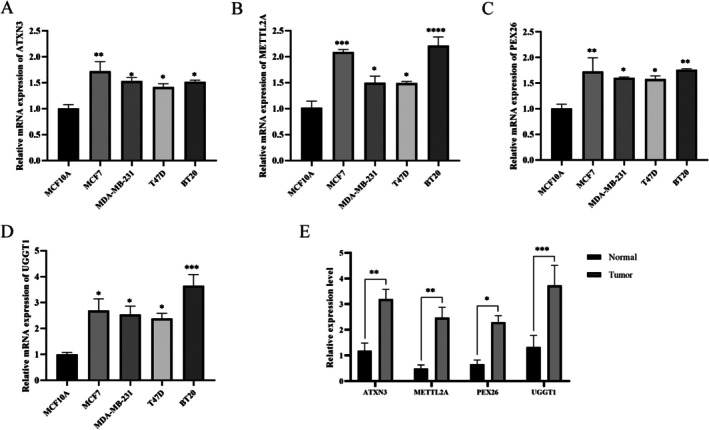
Expression levels of ATIRE in cell lines and tissues. (A–D) The expression levels of ATXN3, METTL2A, PEX26 and UGGT1 in normal breast epithelial cells and breast cancer cells were detected by RT‐qPCR. (E) The expression levels of ATXN3, METTL2A, PEX26 and UGGT1 in breast cancer tissues and adjacent paracancerous tissues were detected by RT‐qPCR. (*p < 0.05, **p < 0.01, ***p < 0.001, ****p < 0.001).

ATIRE regulates physiological and pathological processes mainly by affecting the expression of corresponding genes [6, 7]. Among the ATIREs screened with different expression levels, LOC100130744 was identified as an ncRNA. ATXN3, METTL2A, PEX26 and UGGT1 were selected for experimental verification. RT‐qPCR results showed that their expression levels were significantly higher in BRCA cell lines than in normal mammary epithelial cells (*p* < 0.05) (Figure 8A–D). Similarly, their expression was elevated in BRCA tissues compared to adjacent normal tissues (*p* < 0.05) (Figure 8E).

### 3.9 Immunohistochemical Staining

**FIGURE 9:**
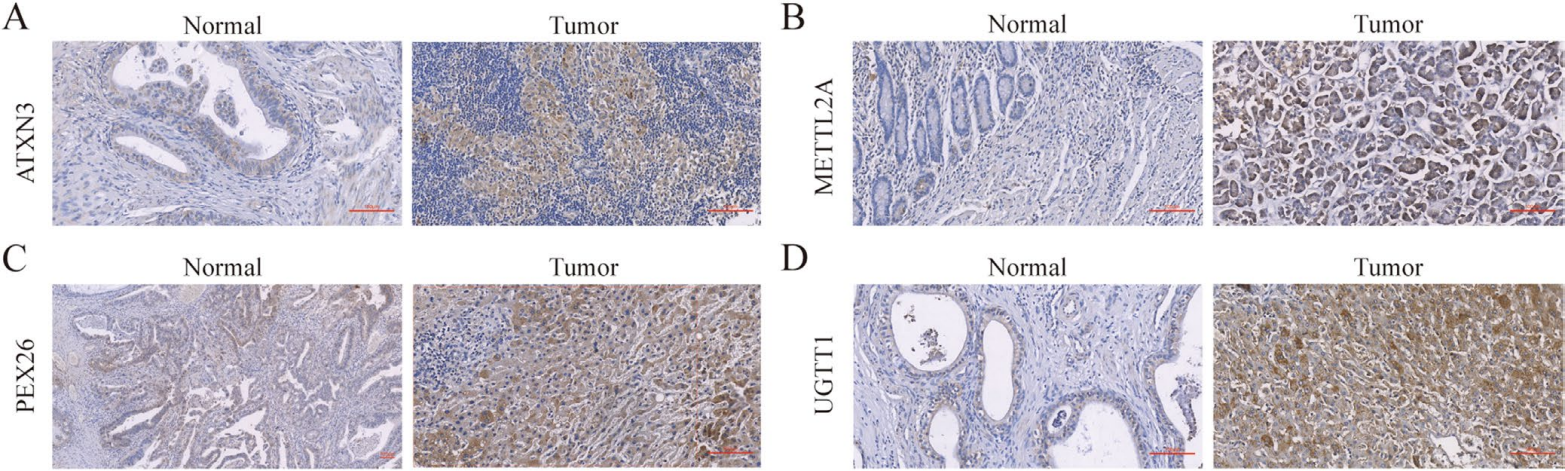
(A–D) Representative immunohistochemical staining in breast cancer and adjacent non‐tumour tissues.

Immunohistochemical analysis revealed significantly higher protein expression levels of ATXN3, METTL2A, PEX26 and UGGT1 in BRCA tissues than in adjacent normal tissues (Figure 9A–D).

## 4 Discussion

RNA editing is an important epigenetic modification that occurs at the RNA level [8]. Its essence is the modification of post‐transcriptional RNA, thereby increasing the diversity of the transcriptome or proteome, and may change the encoded protein sequence or RNA stability and transport. ATIRE is abnormally upregulated in most tumours, and many sites are significantly associated with the pathogenesis of different tumour types [9, 10, 11, 12, 13].

In this study, nine ATIRE sites associated with BRCA survival were identified after Cox and Lasso regression analyses of BRCA data in the TCGA database and were used to construct a prognostic model. Survival analysis revealed that patients in the high‐risk group had worse OS, higher mortality, worse tumour T stage, and more RNA editing than those in the low‐risk group, suggesting that ATIRE may lead to poor outcomes in patients with cancer. The nomogram showed that age, tumour grade, and risk score could be independent prognostic factors for patients with BC. The ROC curve and DCA verified that the nomogram and risk model had moderate accuracy in predicting the OS of BRCA, showing a better overall net benefit than stage and age. Functional enrichment analysis revealed that the DEGs were active in immune‐related biological functions, such as immunoglobulin, immune response, and immunodeficiency.

In addition, the expression level of ADAR in tumour tissues increased with elevated risk scores. The adenosine deaminases (RNA) family includes three proteins: ADAR1, ADAR2 and ADAR3 [14]. ADAR1 and ADAR2 have catalytic activities for ATIRE, whereas ADAR3 has an inhibitory effect [15, 16, 17]. High‐throughput sequencing studies indicate that the expression of ADAR1 is elevated in BRCA [18, 19, 20], and gene mutations caused by its mediated RNA editing lead to reduced production of interferons and other inflammatory mediators and promote tumour immune escape [21]. In addition, we observed statistically significant differences in the editing levels of ATXN3 | chr14:92526779, LOC100130744 | chr5:14716392, METTL2A | chr17:60528133, PEX26 | chr22:18572607, and UGGT1 | chr2:128949284 between BRCA tumours and normal tissues, suggesting that aberrant editing of these ATIRE sites may be associated with BRCA development. We performed in vitro experiments using the differentially expressed ATIRE. The expression levels of ATXN3, METTL2A, PEX26 and UGGT1 in tumour cells were higher than those in normal mammary epithelial cells, and their expression in BRCA tissues was higher than that in normal adjacent tissues. These in vitro findings were consistent with the bioinformatic analysis results.

ATXN3 is a deubiquitinating enzyme (DUB) that is ubiquitously expressed in various cell types, including peripheral tissues and neuronal cells, and participates in multiple cellular pathways [22]. It has been primarily studied for its role in neurodegenerative diseases, such as spinocerebellar ataxia type 3 (SCA3/MJD). Recent studies have indicated that ATXN3 plays a dual role in cancer (promoting or inhibiting), depending on the type of cancer and its molecular background. In BRCA, ATXN3 is significantly upregulated and promotes tumour metastasis and invasion by regulating KLF4. Additionally, elevated ATXN3 expression enhanced carboplatin resistance in BRCA cells. Clinically, high ATXN3 levels are associated with poor patient prognosis [23]. In cervical cancer and colorectal cancer, downregulation of ATXN3 suppresses tumour cell apoptosis, thereby exerting tumour‐promoting effects [24, 25]. Conversely, in pancreatic and thyroid cancers, ATXN3 expression is significantly upregulated, promoting malignant phenotypes, including cell proliferation, migration, and invasion [26, 27]. Furthermore, in testicular and non‐small cell lung cancers, ATXN3 facilitates tumorigenesis by inhibiting the transcriptional activity of PTEN, a critical tumour suppressor gene [28, 29]. Methyltransferase‐like 2A (METTL2A), a member of the methyltransferase family, primarily catalyses the methylation of RNA or proteins. METTL2A is upregulated in BRCA; its high expression is related to poor survival outcomes in patients with BRCA and plays the role of an oncogene [30]. PEX26 is located on chromosome 22q11.21 and encodes a 34 kDa peroxisome membrane protein that interacts with PEX1 and PEX6 [31]. PEX26 expression is significantly downregulated in colorectal cancer and affects the malignant biological behaviour of colorectal cancer cells through the Wnt signalling pathway, which is associated with low OS of patients with colorectal cancer and plays a role as a tumour suppressor gene [32]. In addition, Dahabieh et al. have reported that disrupting the peroxisome balance by silencing PEX26 could kill drug‐resistant tumours and delay the acquisition of drug resistance [33]. UGGT1 is a protein glycosyltransferase that can selectively carry out N‐linked glycosylation of high‐mannose glycans on proteins and plays an important role in protein folding and transportation [34]. UGGT1 | chr2:128952084 is present in low‐grade gliomas [35]. These findings provide functional evidence supporting the association between these ATIRE loci and BRCA prognosis.

This study had some limitations. The risk prognostic model was constructed based on public databases and its clinical utility requires further validation through large‐scale prospective studies. In addition, although we experimentally verified the expression patterns of the identified ATIREs in cells and tissues, more in‐depth functional assays and in vivo experiments are required to elucidate the precise mechanisms of RNA editing in BRCA pathogenesis.

## 5 | Conclusion

This study generated an ATIRE risk assessment model related to OS in patients with BRCA for the first time and demonstrated good predictive performance for OS in such patients. The screened RNA editing sites will be new prognostic markers for BRCA. An in‐depth exploration of how to reduce adverse RNA editing events related to tumours will help provide new ideas for individualised prognostic assessment and medication guidance for patients. The shortcomings of this study are that it is based on public databases. In addition to requiring a large amount of prospective data to verify the practical application of this model in clinical practice, functional experiments are needed to deeply explore the specific mechanisms of RNA editing in BRCA.

## Author Contributions

**Yangyang Zhang:** conceptualization (equal), software (equal), writing – original draft (equal). **Jia Zhou:** data curation (equal), formal analysis (equal). **Hong Li:** data curation (equal), formal analysis (equal). **Dahai Chai:** investigation (equal). **Bin Lian:** investigation (equal). **Yaobang Liu:** software (equal), writing – original draft (equal). **Li Guo:** investigation (equal), supervision (equal). **Jinping Li:** conceptualization (equal), funding acquisition (equal), writing – review and editing (equal).

## Ethics Statement

The authors have nothing to report.

## Consent

All authors have approved the submission to this journal and the final version of the manuscript.

## Conflicts of Interest

The authors declare no conflicts of interest.

## Supporting information


**Appendix S1:** HALLMARK pathway enrichment analysis of DEGs.

## Data Availability

The data and materials that support these findings are available from the corresponding authors upon reasonable request.
