# Supplementary figures and images for: Nomogram Based on A‐To‐I RNA Editing for Predicting Overall Survival in Patients With Breast Cancer
Source: J Cell Mol Med. 2025 Sep 25;29(18):e70781. doi: 10.1111/jcmm.70781 (PMC12461418; doi:10.1111/jcmm.70781)

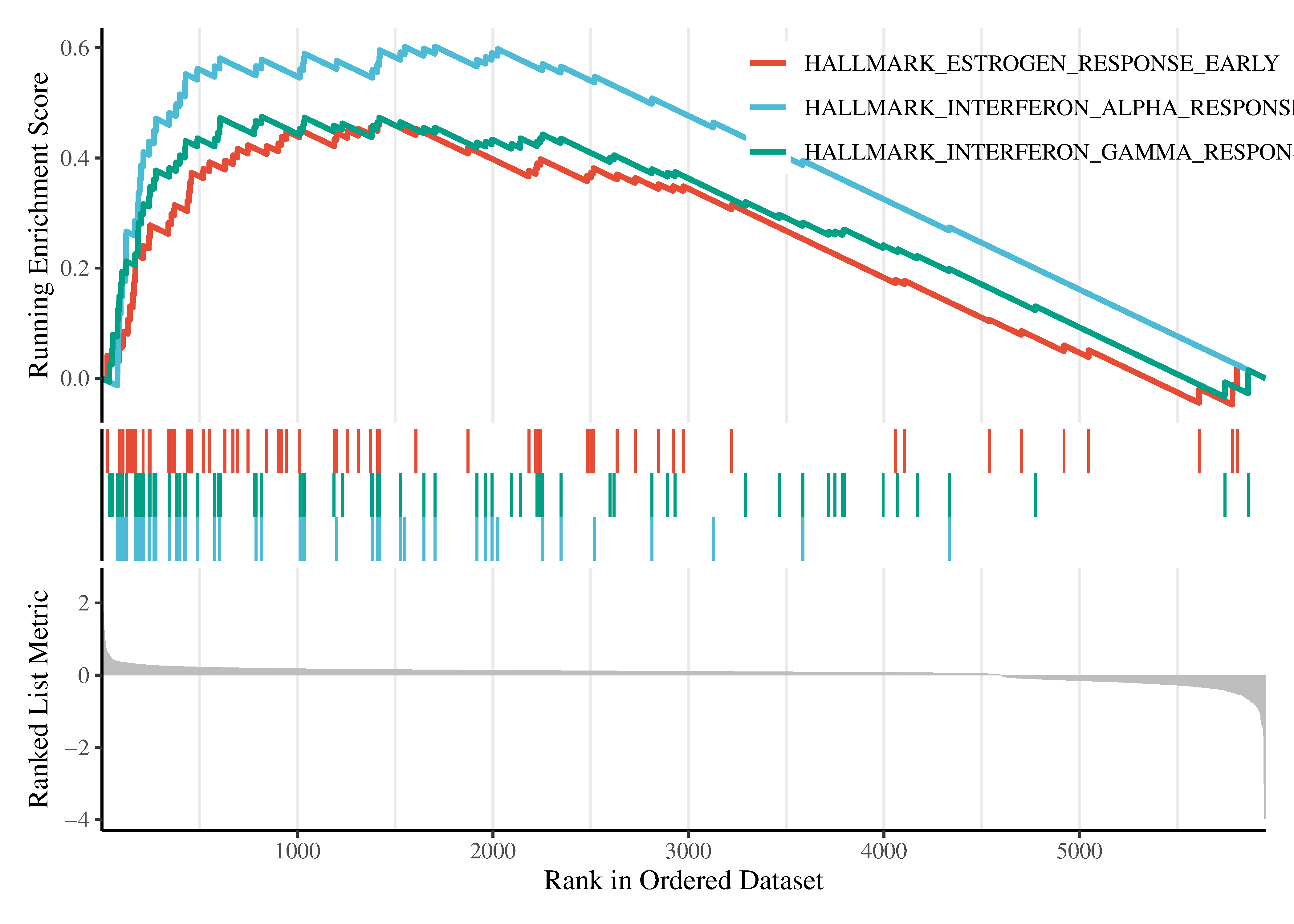

Supplement: Supplementary file 1 — Appendix S1: HALLMARK pathway enrichment analysis of DEGs. [file JCMM-29-e70781-s001.tiff]
